# Clinical manifestations and associated factors of uveitis in patients with pulmonary sarcoidosis: a case control study

**DOI:** 10.1038/s41598-023-49894-5

**Published:** 2023-12-16

**Authors:** Jang Ho Lee, Ye Eun Han, Jiyoul Yang, Ho Cheol Kim, Junyeop Lee

**Affiliations:** 1 Division of Pulmonology and Critical Care Medicine, Department of Internal Medicine, Asan Medical Centre, University of Ulsan College of Medicine, 88 Olympic-ro 43-gil, Songpa-gu, Seoul, 05505 Republic of Korea; 2 Department of Ophthalmology, Asan Medical Center, University of Ulsan College of Medicine, 88 Olympic-ro 43-gil, Songpa-gu, Seoul, 05505 Republic of Korea

**Keywords:** Diseases, Medical research, Risk factors, Signs and symptoms

## Abstract

Sarcoidosis, an idiopathic and inflammatory disease, affects various organs and can manifest as uveitis. Due to limited evidence, researchers investigated the risk factors associated with uveitis in patients with pulmonary sarcoidosis. A retrospective study was conducted on 71 pulmonary sarcoidosis patients, including 19 with uveitis and 52 without. Data on involved organs, imaging findings, spirometry, and analyses from blood and bronchoalveolar lavage fluid were collected. Logistic regression models were used for multivariate analysis. Among the 71 newly diagnosed pulmonary sarcoidosis patients, uveitis was observed in 19 patients (26.8%). No significant differences were found in clinical characteristics between patients with and without uveitis. Fewer patients with uveitis presented lung parenchymal lesions (*P* = 0.043). In multivariate analysis, skin lesions (aOR 7.619, 95% CI 1.277–45.472, *P* = 0.026) and ophthalmic symptoms (aOR 4.065, 95% CI 1.192–13.863, *P* = 0.025) were associated with uveitis. Absence of uveitis was related to lung parenchymal lesions (aOR 0.233, 95% CI 0.062–0.883, *P* = 0.032). Approximately one-quarter of patients with an initial diagnosis of pulmonary sarcoidosis were diagnosed with uveitis. Presence of skin lesions, ophthalmic symptoms, and absence of lung parenchymal lesions were related to uveitis. These results need to be clarified by further studies to confirm the clinical role of early ophthalmologic screening for pulmonary sarcoidosis patients with these factors.

## Introduction

Sarcoidosis is a rare inflammatory disease characterized by noncaseating granulomas in biopsy tissue^1,2^. Although the etiology of sarcoidosis remains unknown, the disease can involve multiple organs and have various manifestations. Sarcoidosis affects 2 to 160 per 100,000 individuals worldwide^3^. The lungs and intrathoracic mediastinal lymph nodes are the most commonly affected organs in sarcoidosis, occurring in more than 90% of patients^3^.

Extrapulmonary organs can also be involved in sarcoidosis. Ocular sarcoidosis, specifically uveitis, is the second most common extrathoracic manifestation, occurring in 25–60% of patients with sarcoidosis^4,5^. Uveitis is the most prevalent form of ocular sarcoidosis^2^, and approximately 30% of sarcoidosis patients experience uveitis during the course of the disease^6^. Undiagnosed and untreated uveitis can result in blindness and a significant decline in quality of life. Consequently, current clinical guidelines for sarcoidosis emphasize the importance of early ophthalmologic screening for prompt detection and timely treatment of uveitis^7,8^. Identifying predictive factors for sarcoidosis-associated uveitis could help clinicians determine the appropriate timing for ophthalmologist referrals. However, few studies have investigated uveitis associated factors in pulmonary sarcoidosis patients. In this study, we aimed to examine the baseline clinical characteristics and associated factors for uveitis in pulmonary sarcoidosis within the Korean population.

## Results

### Study flow and baseline characteristics of the study population

**Figure 1:**
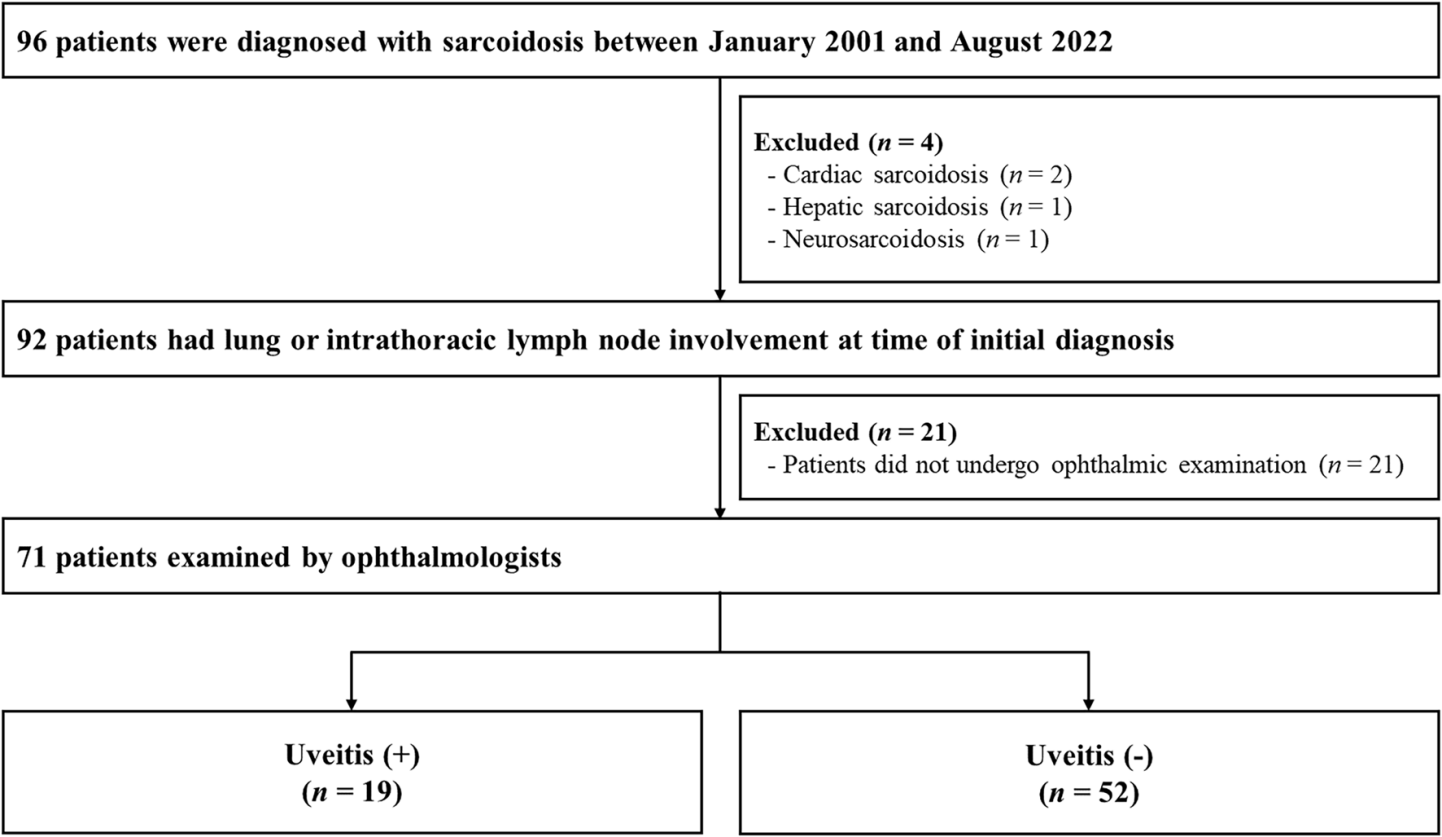
Study flow.

During the study period, 96 patients were diagnosed with sarcoidosis, of which 92 (95.8%) exhibited lung or intrathoracic lymph node involvement at the time of initial diagnosis. Out of these patients, 71 (77.2%) underwent ophthalmologic examinations to investigate ophthalmic manifestations and were included in the final analysis. Based on the ophthalmic exam results, we divided them into uveitis and non-uveitis groups (Fig. 1). The baseline characteristics of the study population are detailed in Table 1. Among the 71 patients, 19 (26.8%) were diagnosed with uveitis, while 52 (73.2%) were not. The two groups showed no significant differences except for accompanying ophthalmic symptoms. Patients with uveitis were more likely to have ophthalmic symptoms (such as floaters, injection, ocular pain, and decreased vision) than non-uveitis patients (*n* = 12, 63.2% vs.* n* = 18, 34.6%).

**Table 1:** Baseline characteristics of the study population.

	Total	Uveitis group	Non-uveitis group	*P*-value
	(*n* = 71)	(*n* = 19)	(*n* = 52)	
Sex				0.734
Male	35 (49.3%)	10 (52.6%)	25 (48.1%)	
Female	36 (50.7%)	9 (47.4%)	27 (51.9%)	
Age, yrs	49.7 ± 13.3	51.4 ± 11.8	49.1 ± 13.8	0.631
Body mass index, kg/m^2^	24.8 ± 3.7	23.8 ± 2.9	25.1 ± 3.9	0.112
Smoking history				0.787
Ever smoker	28 (39.4%)	7 (36.8%)	21 (40.4%)	
Never smoker	43 (60.6%)	12 (63.2%)	31 (59.6%)	
Smoking amount, pack*yrs	6.1 ± 12.2	4.6 ± 8.7	6.6 ± 13.3	0.691
Comorbidities				
Diabetes mellitus*	10 (14.1%)	1 (5.3%)	9 (17.3%)	0.270
Cardiovascular disease*	15 (21.1%)	3 (15.8%)	12 (23.1%)	0.744
Respiratory disease*	3 (4.2%)	1 (5.3%)	2 (3.8%)	> 0.999
Ophthalmic symptom	30 (42.3%)	12 (63.2%)	18 (34.6%)	0.031

Data are presented as mean ± standard deviation or frequencies (%). Ophthalmic symptoms were investigated at the initial diagnosis of sarcoidosis, to evaluate the association between baseline ophthalmic symptoms and existence of uveitis. Difference between both groups was analyzed by the chi-square test or Fisher exact test for categorical variables and Mann–Whitney U test for continuous variables. Asterix (*) indicates variables analyzed by Fisher exact test.

Table 2 presents the involved organs and laboratory findings. Lung involvement was more common in the non-uveitis group (86.5% in the non-uveitis group *vs.* 63.2% in the uveitis group, *P*-value = 0.043). Conversely, more patients in the uveitis group had sarcoidosis involvement in the musculoskeletal system. There were no significant differences in laboratory findings between the two groups. Of the 71 enrolled patients, 66 underwent pulmonary function tests (18 patients in the uveitis group and 48 in the non-uveitis group). The results of the pulmonary function tests are presented in Supplementary Table S1, with no statistically significant differences in any variable.

**Table 2:** Organ involvement and laboratory findings in the study population.

	Total	Uveitis group	Non-uveitis group	*P*-value
	(*n* = 71)	(*n* = 19)	(*n* = 52)	
Pulmonary sarcoidosis*				0.043
LN only	14 (19.7%)	7 (36.8%)	7 (13.5%)	
Lung ± Intrathoracic LN	57 (80.3%)	12 (63.2%)	45 (86.5%)	
Other involved organs				
Extrathoracic LN*	13 (18.3%)	5 (26.3%)	8 (15.4%)	0.313
Skin*	7 (9.9%)	4 (21.1%)	3 (5.8%)	0.077
Spleen*	6 (8.5%)	1 (5.3%)	5 (9.6%)	> 0.999
Heart*	5 (7.0%)	2 (10.5%)	3 (5.8%)	0.605
Musculoskeletal*	5 (7.0%)	5 (26.3%)	0 (0.0%)	0.001
Nervous system*	3 (4.2%)	2 (10.5%)	1 (1.9%)	0.173
Kidney*	1 (1.4%)	1 (5.3%)	0 (0.0%)	0.268
Serum ACE level (U/L)	60.7 ± 27.9	65.9 ± 29.8	58.8 ± 27.3	0.215
Serum corrected calcium (mg/dL)	9.7 ± 0.4	9.7 ± 0.4	9.7 ± 0.4	0.428
Serum creatinine level (mg/dL)	0.9 ± 0.3	0.8 ± 0.2	0.9 ± 0.3	0.706
Serum alkaline phosphatase (IU/L)	78.2 ± 37.5	84.7 ± 48.4	75.8 ± 32.9	0.432
Serum white blood cell (/uL)	6223.9 ± 1951.7	6247.4 ± 2260.9	6215.4 ± 1850.4	0.663
Serum lymphocyte level (/uL)	1723.4 ± 722.6	1601.3 ± 844.0	1768.1 ± 676.5	0.356
Serum lymphopenia	31 (43.7%)	10 (52.6%)	21 (40.4%)	0.357
Serum hemoglobin (g/dL)	13.6 ± 1.7	13.7 ± 1.2	13.6 ± 1.9	0.677
Serum platelet (× 10^3^/uL)	257.6 ± 76.1	274.4 ± 79.1	251.5 ± 74.8	0.116

Data are presented as mean ± standard deviation or frequencies (%). Difference between both groups was analyzed by the chi-square test or Fisher exact test for categorical variables and Mann–Whitney U test for continuous variables. Asterix (*) indicates variables analyzed by Fisher exact test.

*ACE*, angiotensin-converting enzyme; *LN*, lymph node.

### Ophthalmic examination results

**Figure 2:**
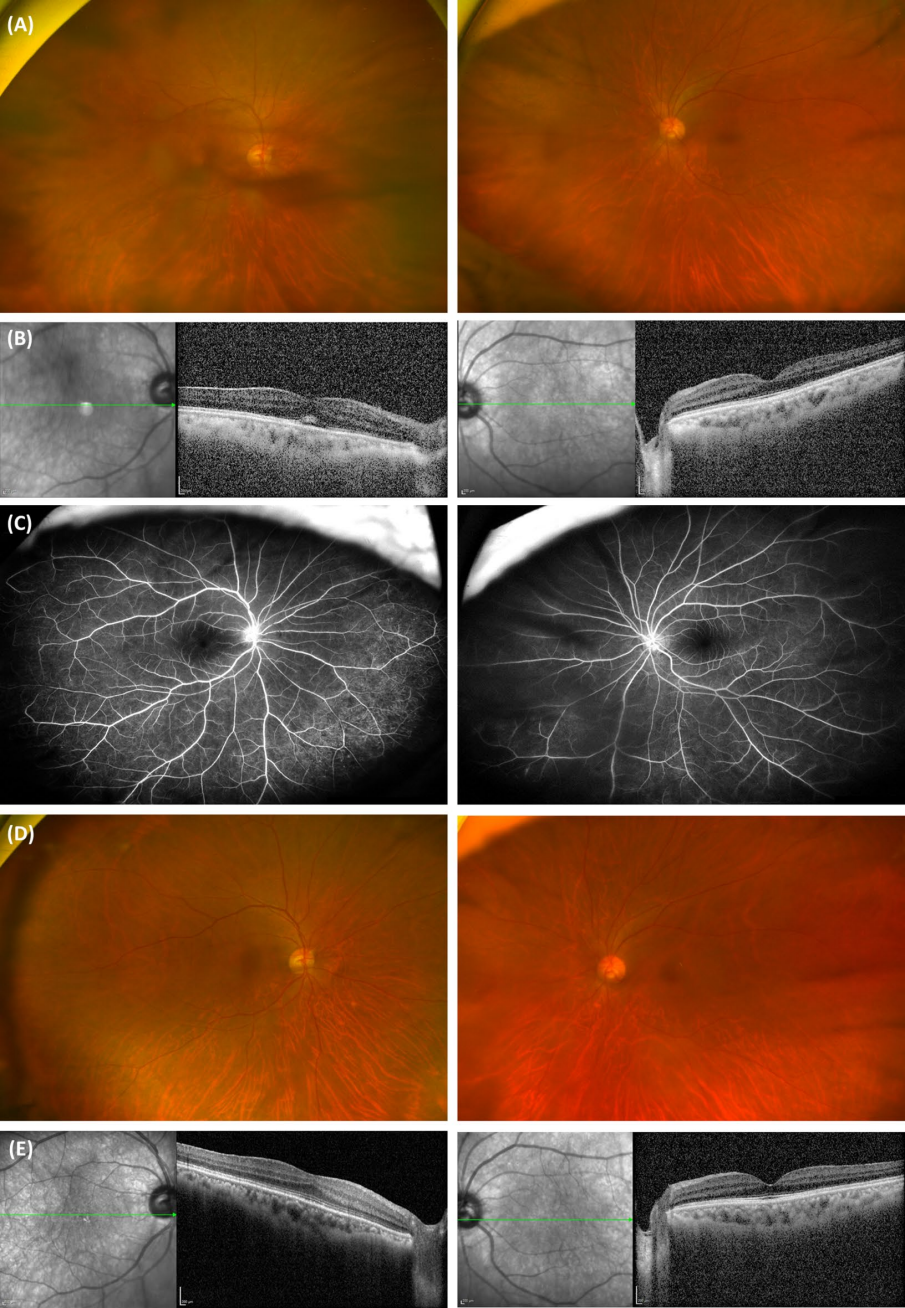
A representative case of sarcoidosis-associated uveitis. A 67-year-old female patient with pulmonary sarcoidosis and skin involvement reported decreased vision (right 0.3 and left 0.5 in Snellen). Clinical manifestations of uveitis, including vitreous opacity and retinal infiltrations, were evident in wide-field fundus photography (**A**). Subretinal fluid in the right eye and vitreous haze and cells in both eyes were noted in optical coherence tomography (**B**). Diffuse vascular staining, leakage, and Dalen-Fuchs nodule-like lesions, which indicated retinal vasculitis and inflammation, were observed in wide-field fluorescein angiography (**C**). After five months of topical and oral prednisolone treatments and a 13-month follow-up period, ocular inflammation signs improved in fundus photography and OCT (**D**, **E**). The patient’s visual acuity also improved to 0.6 in the right eye and remained at 0.5 in the left eye on the Snellen chart.

The types of uveitis observed were anterior (inflammation in the iris and ciliary body, *n* = 2, 10.5%), posterior (inflammation in the retina and choroid, *n* = 7, 36.8%), and pan-uveitis (inflammation in the entire uveal tissue, *n* = 10, 52.6%), with all cases being bilateral. Among the posterior uveitis cases, 5 (71.4%) displayed subclinical inflammation, with uveitis signs detectable only through angiographic evaluation. Treatments for uveitis included topical prednisolone (1%, 2 to 8 times/day) for all types of uveitis based on the severity of inflammation, as well as oral prednisolone 1 mg/kg/day with tapering and subtenon triamcinolone injection as needed for posterior and pan-uveitis. All types of uveitis exhibited complete resolution, while 47% (*n* = 8) of posterior and pan-uveitis patients had mild vitreous opacity without active inflammatory activity. A representative case of sarcoidosis-associated uveitis was presented in Fig. 2.

### Univariate and multivariate analysis for uveitis

We performed univariate and multivariate analyses using variables that demonstrated a difference with a *P*-value less than 0.1 (Table 3). The included variables were ophthalmic symptoms, lung parenchymal involvement, and skin involvement. In the univariate analysis, lung parenchymal involvement (OR 0.267, 95% CI 0.078–0.909, *P*-value = 0.035) and ophthalmic symptoms (OR 3.238, 95% CI 1.085–9.663, *P*-value = 0.035) were identified as significant factors related to uveitis at the first ophthalmic examination following sarcoidosis diagnosis. In the multivariate analysis, all included variables were significantly associated with uveitis (lung parenchymal involvement: aOR 0.233, 95% CI 0.062–0.883, *P*-value = 0.032; skin involvement: aOR 7.619, 95% CI 1.277–45.472, *P*-value = 0.026; and ophthalmic symptoms: aOR 4.065, 95% CI 1.192–13.863, *P*-value = 0.025).

**Table 3:** Univariate and multivariate analysis of factors associated with uveitis in pulmonary sarcoidosis.

Covariates	Univariate analysis		Multivariate analysis	
	OR	*P*-value	aOR	*P*-value
Lung lesion	0.267 (0.078–0.909)	0.035	0.233 (0.062–0.883)	0.032
Skin lesion	4.356 (0.875–21.677)	0.072	7.619 (1.277–45.472)	0.026
Ophthalmic symptom	3.238 (1.085–9.663)	0.035	4.065 (1.192–13.863)	0.025

Logistic regression models were used for univariate and multivariate analyses. In these analyses, ophthalmic symptoms and skin lesions were associated with increased uveitis in pulmonary sarcoidosis patients, while lung lesions at initial diagnosis were related to the absence of uveitis lesions in the initial ophthalmic examination. Goodness of fit for logistic regression was investigated by Hosmer–Lemeshow test (*P* values = 0.505).

*OR*, odds ratio.

### Baseline characteristics of patients with bronchoalveolar lavage

We conducted a subgroup analysis of 48 patients who underwent bronchoalveolar lavage (11 patients in the uveitis group and 37 patients in the non-uveitis group). When comparing baseline characteristics, involved organs, laboratory findings, and bronchoalveolar lavage fluid analysis, no variable showed a significant difference between the two groups (Supplementary Table S2 and S3). Only the presence of more than 30% lymphocytes in bronchoalveolar lavage exhibited a difference between the groups with a *P*-value less than 0.1. A higher proportion of lymphocytes (more than 30%) in bronchoalveolar lavage was associated with a higher frequency of uveitis in the univariate analysis (OR = 4.263, 95% CI 0.809–22.472, *P*-value = 0.087); however, this finding was not statistically significant.

## Discussion

In clinical practice, the presence of uveitis in patients with sarcoidosis has been a subject of investigation. While one study suggested that race and sex influenced the manifestation of uveitis, there has been limited evidence regarding its associated factors^9^. In our study, approximately one-fourth of the study population was diagnosed with uveitis, which is consistent with previously reported prevalence rates in biopsy-proven sarcoidosis cases^10–13^. We found that skin involvement and accompanying ophthalmic symptoms were associated with the presence of uveitis in patients with pulmonary sarcoidosis at the time of initial diagnosis. In contrast, lung parenchymal involvement at the initial diagnosis was related to a lower frequency of uveitis. To our knowledge, this is the first study to investigate factors associated with uveitis at the initial diagnosis of pulmonary sarcoidosis.

Skin involvement has been reported in 3.0–42.8% of sarcoidosis patients^14,15^. In our study, skin involvement was identified as a factor associated with uveitis. A similar result was reported in a study conducted by Schupp et al.^16^ The authors classified the phenotypes of organ involvement in sarcoidosis and identified the ocular-cardiac-cutaneous-central nervous system phenotype as one of the subgroups. In their study, patients with ocular involvement had a higher rate of skin, heart, and central nervous system involvement. Although not statistically significant, a similar trend was observed in our study.

The musculoskeletal system was the only organ significantly more involved in sarcoidosis within the uveitis group. In the aforementioned study, patients with musculoskeletal and cutaneous involvement were more affected by ocular involvement^16^. These findings suggest that differences in organ involvement in sarcoidosis could stem from distinct pathogenesis in individual patients. However, no studies have yet elucidated this point.

In this study, we found that the presence of ophthalmic symptoms was associated with uveitis, highlighting the fact that sarcoidosis patients experiencing these symptoms should be promptly referred to an ophthalmologist. Although the rate of symptom complaints among uveitis patients in our study was higher than in previous research^17–19^, only about half of these patient-reported symptoms. Moreover, the reported symptoms were variable and did not correlate with the severity of inflammation. This finding suggests that early ophthalmic examinations are crucial for sarcoidosis patients, regardless of the presence of ophthalmic symptoms. Notably, approximately 70% of patients with posterior uveitis exhibited subclinical vasculitis, which was only detectable through angiographic evaluation. We recommend wide-field fluorescein angiography for sarcoidosis patients, particularly those presenting with signs of inflammation or ophthalmic symptoms, to enhance the detection of peripheral vasculitis or disc inflammation and improve diagnostic accuracy.

Our study also revealed that the presence of lung parenchymal lesions was associated with a lower incidence of uveitis. Mediastinal lymph node involvement without lung parenchymal lesions could potentially delay the diagnosis of sarcoidosis. In a previous study on cutaneous sarcoidosis, lung parenchymal lesions were more prevalent in the non-cutaneous involvement group compared to the cutaneous involvement group^20^. Delayed diagnosis might lead to postponed treatment, allowing systemic sarcoidosis involvement to progress, including ocular or cutaneous manifestations. Therefore, even in the absence of lung parenchymal lesions, patients diagnosed with sarcoidosis involving lymph nodes may benefit from ophthalmic screening examinations for uveitis.

Several studies have reported an association between an increased lymphocyte proportion in bronchoalveolar lavage fluid analysis and the presence of uveitis^21–23^. In our study, although not statistically significant, patients with more than 30% lymphocytes in bronchoalveolar lavage fluid analysis were more common in the uveitis group. Takahashi et al. investigated bronchoalveolar lavage fluid analysis in 39 patients with suspected ocular sarcoidosis^23^. In their study, 23 patients (59.0%) showed more than 30% lymphocytes in bronchoalveolar lavage, a proportion relatively lower than our findings. This discrepancy may result from the lower proportion of patients with lung parenchymal lesions in chest computed tomography in Takahashi et al.’s study (20 of 39 patients, 51.3%) compared to ours (9 of 11 patients, 81.8%). Previous studies have reported that a higher proportion of lymphocytes in bronchoalveolar lavage fluid analysis reflects the activity and severity of sarcoidosis^24–26^. Additionally, several forms of extrathoracic sarcoidosis are accompanied by lymphocytosis in bronchoalveolar lavage^23,27,28^. Due to the relatively small number of pulmonary sarcoidosis cases investigated, further studies are needed to evaluate the relationship between uveitis presence and more than 30% lymphocytes in bronchoalveolar lavage fluid analysis.

This study has several limitations. First, it involved a retrospective review of clinical data and sarcoidosis-related information, and the patient sample size was relatively small. We selected variables for logistic regression based on the statistical result rather than clinical implication, due to lack of studies reporting risk factors related to uveitis. This could lead to overlook association of unreported clinically important factor with uveitis. To obtain clearer results, prospective studies with larger cohorts may be necessary. Second, our findings may not be generalizable to all types of sarcoidosis since we only included Korean pulmonary sarcoidosis patients. However, more than 90% of sarcoidosis patients have pulmonary or mediastinal lymph node involvement. Furthermore, all patients had only uveitis, without other types of ocular sarcoidosis. Finally, we could not perform angiographic analysis for the entire study population due to its retrospective nature. The proportion of sarcoidosis patients with uveitis may have been higher if angiographic evaluations had been conducted for all patients.

In patients with an initial diagnosis of pulmonary sarcoidosis, approximately 27% were diagnosed with uveitis. Accompanied skin lesions, ophthalmic symptoms, and the absence of lung parenchymal lesions were reported as associated factors for uveitis. These results need to be clarified by further studies to confirm the clinical role of early ophthalmologic screening for pulmonary sarcoidosis patients with these factors.

## Methods

### Study design and participants

We conducted a single-center retrospective study at Asan Medical Center in the Republic of Korea. We screened the patients included in the list of sarcoidosis diagnosis between January 2001 and August 2022. Inclusion criteria were as follows: (1) sarcoidosis diagnosis based on the World Association of Sarcoidosis and Other Granulomatous Disease diagnostic criteria, which includes noncaseating granuloma on biopsy tissue, compatible symptoms, and exclusion of other possible diseases^29^, (2) lung parenchyma and/or mediastinal lymph node involvement confirmed by biopsy, and (3) ophthalmic examination reviewed to investigate the presence of uveitis. We excluded patients with no history of ophthalmic examination at the initial diagnosis of pulmonary sarcoidosis or no evidence for lung parenchyma and/or mediastinal lymph node involvement.

The Institutional Review Board of Asan Medical Center (IRB No. 2021–1811) approved this study and waived the requirement for informed consent due to the retrospective nature of the analysis. This study was conducted following the amended Declaration of Helsinki.

### Measurements

Sarcoidosis-associated uveitis was diagnosed based on the revised International Workshop on Ocular Sarcoidosis Criteria^30^. To assess ocular status, all referred patients underwent a thorough ophthalmic examination on the referral day, including slit-lamp biomicroscopy, funduscopic examination, and spectral-domain optical coherence tomography (Heidelberg Engineering GmbH; Heidelberg, Germany) by retinal specialists (YEH and JL). Additional angiographic evaluation using wide-field fluorescein angiography (Optos California: Optos plc, Dunfermline, United Kingdom) was conducted, particularly for those with suspicion but no definitive signs of uveitis (e.g., mild vitreous opacity, mild vascular abnormalities).

Baseline characteristics were reviewed using electronic medical records. Demographic characteristics, including sex, age, body mass index, smoking history, comorbidities, and accompanying ophthalmic symptoms at initial diagnosis, were investigated. To evaluate the state related to underlying sarcoidosis, we examined laboratory findings and involved organs. As it was not possible to confirm the pathologic diagnosis for all suspicious lesions, we judged the involved organs based on radiologic studies. Although not all patients underwent the tests, we investigated the results of pulmonary function tests, six-minute walk tests, and bronchoalveolar lavage performed before the initial sarcoidosis diagnosis or during hospitalization for diagnosis.

### Statistical analysis

All data are presented as numbers (percentage) and mean ± standard deviation for categorical and continuous variables, respectively. Categorical variables were compared using the χ2 or Fisher’s exact test. Differences in continuous variables were analyzed using Mann–Whitney U test. We utilized Logistic regression analysis to investigate associated factors with uveitis. Independent variables were selected based on statistical significance (*P*-value < 0.1) for univariate and multivariate analyses. Logistic regression analysis with backward selection was used for univariate and multivariate analyses to calculate odds ratios (OR) with 95% confidence intervals (CI). All tests of significance were two-sided, and a *P*-value of < 0.05 was considered indicative of statistical significance. As bronchoalveolar lavage was performed only in a subset of enrolled patients, we conducted a subgroup analysis with patients who received bronchoalveolar lavage at the initial time of diagnosis. All analyses were performed using SPSS software (version 24.0; Chicago, IL, USA).

### Supplementary Information

Supplementary Tables.

## Data Availability

The datasets used and/or analyzed during this study can be made available by the corresponding author upon reasonable request.

## Abbreviations

CI: Confidence interval
OR: Odds ratio

## References

[1] Drent M, Crouser ED, Grunewald J (2021). Challenges of sarcoidosis and its management. N. Engl. J. Med..

[2] Jamilloux Y, Kodjikian L, Broussolle C, Seve P (2014). Sarcoidosis and uveitis. Autoimmun. Rev..

[3] Belperio JA (2022). Diagnosis and treatment of pulmonary sarcoidosis: A review. JAMA.

[4] Pasadhika S, Rosenbaum JT (2015). Ocular sarcoidosis. Clin Chest Med..

[5] Ungprasert P, Ryu JH, Matteson EL (2019). Clinical manifestations, diagnosis, and treatment of sarcoidosis. Mayo Clin. Proc. Innov. Qual. Outcomes.

[6] Bodaghi B, Touitou V, Fardeau C, Chapelon C, LeHoang P (2012). Ocular sarcoidosis. Presse Med..

[7] Crouser ED (2020). Diagnosis and detection of sarcoidosis. An official American thoracic society clinical practice guideline. Am. J. Respir. Crit. Care Med..

[8] Baughman RP (2021). ERS clinical practice guidelines on treatment of sarcoidosis. Eur. Respir. J..

[9] Rothova A (1989). Risk factors for ocular sarcoidosis. Doc. Ophthalmol Adv. Ophthalmol..

[10] Sungur G, Hazirolan D, Bilgin G (2013). Pattern of ocular findings in patients with biopsy-proven sarcoidosis in Turkey. Ocul. Immunol. Inflamm..

[11] Evans M, Sharma O, LaBree L, Smith RE, Rao NA (2007). Differences in clinical findings between Caucasians and African Americans with biopsy-proven sarcoidosis. Ophthalmology.

[12] Choi SY, Lee JH, Won J-Y, Shin JA, Park Y-H (2018). Ocular manifestations of biopsy-proven pulmonary sarcoidosis in Korea. J. Ophthalmol..

[13] Radosavljević A (2017). Clinical features of ocular sarcoidosis in patients with biopsy-proven pulmonary sarcoidosis in Serbia. Ocul. Immunol. Inflammat..

[14] Ying Z (2017). Clinical characteristics of sarcoidosis patients in the United States versus China. Sarcoidosis Vasc. Diffuse Lung Dis..

[15] Te HS (2020). Clinical characteristics and organ system involvement in sarcoidosis: comparison of the University of Minnesota Cohort with other cohorts. BMC Pulm. Med..

[16] Schupp JC (2018). Phenotypes of organ involvement in sarcoidosis. Eur. Respir. J..

[17] Heiligenhaus A, Wefelmeyer D, Wefelmeyer E, Rösel M, Schrenk M (2011). The eye as a common site for the early clinical manifestation of sarcoidosis. Ophthalmic Res..

[18] Matsuo T, Fujiwara N, Nakata Y (2005). First presenting signs or symptoms of sarcoidosis in a Japanese population. Jpn. J. Ophthalmol..

[19] Lee J (2022). The role of screening for asymptomatic ocular inflammation in sarcoidosis. Ocul. Immunol. Inflammat..

[20] Yanardag H, Pamuk ON, Karayel T (2003). Cutaneous involvement in sarcoidosis: analysis of the features in 170 patients. Respir. Med..

[21] Caspers LE, Ebraert H, Makhoul D, Willermain F, Michel O (2014). Broncho-alveolar lavage (BAL) for the diagnosis of sarcoidosic uveitis. Ocul. Immunol. Inflamm..

[22] Sugimoto M, Nakashima H, Ando M, Kohrogi H, Araki S (1989). Bronchoalveolar lavage studies in uveitis patients without radiological intrathoracic involvement of sarcoidosis. Jpn. J. Med..

[23] Takahashi T (2001). Significance of lymphocytosis in bronchoalveolar lavage in suspected ocular sarcoidosis. Eur. Respir. J..

[24] Danila E, Jurgauskiene L, Norkuniene J, Malickaite R (2009). BAL fluid cells in newly diagnosed pulmonary sarcoidosis with different clinical activity. Upsala J. Med. Sci..

[25] Ziegenhagen MW, Rothe ME, Schlaak M, Muller-Quernheim J (2003). Bronchoalveolar and serological parameters reflecting the severity of sarcoidosis. Eur. Respir. J..

[26] Kobak S (2020). Catch the rainbow: Prognostic factor of sarcoidosis. Lung India.

[27] Petek BJ (2018). Cardiac sarcoidosis: Diagnosis confirmation by bronchoalveolar lavage and lung biopsy. Respir. Med..

[28] Akaba T (2020). Coexistence of diffuse panbronchiolitis and sarcoidosis revealed during splenectomy: A case report. BMC Pulm. Med..

[29] Hunninghake GW (1999). ATS/ERS/WASOG statement on sarcoidosis. American Thoracic Society/European Respiratory Society/World Association of Sarcoidosis and other Granulomatous Disorders. Sarcoidosis Vasc. Diffuse Lung Dis..

[30] Mochizuki M (2019). Revised criteria of International Workshop on Ocular Sarcoidosis (IWOS) for the diagnosis of ocular sarcoidosis. Br. J. Ophthalmol..

