# Supplementary material for: Clinical manifestations and associated factors of uveitis in patients with pulmonary sarcoidosis: a case control study
Source: Sci Rep. 2023 Dec 16;13:22380. doi: 10.1038/s41598-023-49894-5 (PMC10725472; doi:10.1038/s41598-023-49894-5)
Supplement: Supplementary file 1 — Supplementary Tables. [file 41598_2023_49894_MOESM1_ESM.docx]

Supplementary Table S1. Pulmonary function test results in the study population

|  | Total | Uveitis group | Non-uveitis group | *P*-value |
| --- | --- | --- | --- | --- |
| Pulmonary function test | *n* = 66 | *n* = 18 | *n* = 48 |  |
| FEV_1_ % pred | 85.6 ± 13.8 | 85.6 ± 17.4 | 85.6 ± 12.4 | 0.498 |
| FVC % pred | 88.2 ± 11.7 | 87.5 ± 14.2 | 88.5 ± 10.8 | 0.751 |
| FEV_1_/FVC ratio | 77.6 ± 8.6 | 78.3 ± 11.3 | 77.3 ± 7.4 | 0.330 |
| TLC % pred (*n* = 39) | 89.8 ± 9.7 | 90.3 ± 13.4 | 89.6 ± 8.3 | 0.530 |
| DLCO % pred (*n* = 62) | 78.6 ± 13.9 | 79.7 ± 12.0 | 78.2 ± 14.7 | 0.918 |
| Distant 6MWT (m, *n* = 23) | 521.4 ± 93.4 | 551.5 ± 52.4 | 510.8 ± 103.3 | 0.327 |

Data are presented as mean ± standard deviation or frequencies (%). The following variables were measured only in a subset of the study population: total lung capacity (10 patients in the uveitis group and 29 in the no uveitis group), diffusing capacity (17 patients in the uveitis group and 45 in the no uveitis group), and six-minute walk test (6 patients in the uveitis group and 17 patients in the no uveitis group). Difference between both groups was analyzed by Mann-Whitney U test.

Abbreviations: *6MWT*, six-minute walk test; *DLCO*, diffusing capacity of the lung for carbon monoxide; *FEV_1_*, forced expiratory volume in 1 second; *FVC*, forced vital capacity; *TLC*, total lung capacity.

Supplementary Table S2. Baseline characteristics and bronchoalveolar lavage fluid analysis of patients who received bronchoalveolar lavage

|  | Total | | Uveitis group | | Non-uveitis group | | *P*-value | |  |
| --- | --- | --- | --- | --- | --- | --- | --- | --- | --- |
|  | (*n* = 48) | | (*n* = 11) | | (*n* = 37) | |  |  |  |
| Sex* |  | |  | |  | | 0.731 | |  |
| Male | 24 (50.0%) | | 6 (54.5%) | | 18 (48.6%) | |  | |  |
| Female | 24 (50.0%) | | 5 (45.5%) | | 19 (51.4%) | |  | |  |
| Age, yrs | 48.7 ± 13.9 | | 50.6 ± 13.3 | | 48.1 ± 14.2 | | 0.632 | |  |
| Body mass index, kg/m^2^ | 24.6 ± 4.0 | | 23.3 ± 1.9 | | 25.0 ± 4.4 | | 0.131 | |  |
| Smoking history* |  | |  | |  | | >0.999 | |  |
| Ever smoker | 21 (43.8%) | | 5 (45.5%) | | 16 (43.2%) | |  | |  |
| Never smoker | 27 (56.2%) | | 6 (54.5%) | | 21 (56.8%) | |  | |  |
| Smoking amount, pack*yrs | 7.6 ± 14.1 | | 5.9 ± 9.8 | | 8.1 ± 15.2 | | 0.925 | |  |
| Comorbidities |  | |  | |  | |  | |  |
| Diabetes mellitus* | 8 (16.7%) | | 1 (9.1%) | | 7 (18.9%) | | 0.661 | |  |
| Cardiovascular disease* | 10 (20.8%) | | 1 (9.1%) | | 9 (24.3%) | | 0.416 | |  |
| Respiratory disease* | 2 (4.2%) | | 0 (0.0%) | | 2 (5.4%) | | >0.999 | |  |
| Ophthalmic symptom* | 18 (37.5%) | | 6 (54.5%) | | 12 (32.4%) | | 0.288 | |  |
| Bronchoalveolar lavage | | *n* = 48 | | *n* = 11 | | *n* = 37 | |  | |
| White blood cell | | 163.6 ± 108.3 | | 176.4 ± 119.7 | | 159.8 ± 106.1 | | 0.686 | |
| Neutrophil | | 1.4 ± 2.6 | | 0.7 ± 1.2 | | 1.5 ± 2.9 | | 0.554 | |
| Lymphocyte | | 35.4 ± 21.0 | | 41.3 ± 16.9 | | 33.6 ± 22.0 | | 0.215 | |
| Eosinophil | | 0.6 ± 1.0 | | 0.8 ± 1.3 | | 0.5 ± 0.9 | | 0.401 | |
| Macrophage | | 62.5 ± 21.7 | | 56.9 ± 17.5 | | 64.2 ± 22.7 | | 0.280 | |
| Lymphocyte (>15%)* | | 37 (77.1%) | | 9 (81.8%) | | 28 (75.7%) | | >0.999 | |
| Lymphocyte (>30%)* | | 28 (58.3%) | | 9 (81.8%) | | 19 (51.4%) | | 0.092 | |

Data are presented as mean ± standard deviation or frequencies (%). Difference between both groups was analyzed by the chi-square test or Fisher exact test for categorical variables and Mann-Whitney U test for continuous variables. Asterix (*) indicates variables analyzed by Fisher exact test.

*Fisher exact test.

Supplementary Table S3. Organ involvement and laboratory findings in patients who received bronchoalveolar lavage

|  | Total | Uveitis group | Non-uveitis group | *P*-value |
| --- | --- | --- | --- | --- |
|  | (*n* = 48) | (*n* = 11) | (*n* = 37) |  |
| Pulmonary sarcoidosis* |  |  |  | 0.127 |
| LN only | 3 (6.2%) | 2 (18.2%) | 1 (2.7%) |  |
| Lung ± Intrathoracic LN | 45 (93.8%) | 9 (81.8%) | 36 (97.3%) |  |
| Other involved organs |  |  |  |  |
| Extrathoracic LN* | 8 (16.7%) | 2 (18.2%) | 6 (16.2%) | >0.999 |
| Skin* | 6 (12.5%) | 3 (27.3%) | 3 (8.1%) | 0.124 |
| Spleen* | 3 (6.3%) | 1 (9.1%) | 2 (5.4%) | 0.551 |
| Heart* | 5 (10.4%) | 2 (18.2%) | 3 (8.1%) | 0.321 |
| Musculoskeletal* | 2 (4.2%) | 2 (18.2%) | 0 (0.0%) | 0.049 |
| Nervous system* | 1 (2.1%) | 0 (0.0%) | 1 (2.7%) | >0.999 |
| Kidney* | 1 (2.1%) | 1 (9.1%) | 0 (0.0%) | 0.229 |
| Serum ACE level (U/L) | 63.6 ± 29.5 | 72.1 ± 28.8 | 61.1 ± 29.7 | 0.158 |
| Serum corrected calcium (mg/dL) | 9.6 ± 0.4 | 9.7 ± 0.3 | 9.6 ± 0.4 | 0.668 |
| Serum creatinine level (mg/dL) | 0.8 ± 0.2 | 0.8 ± 0.2 | 0.8 ± 0.2 | 0.951 |
| Serum alkaline phosphatase (IU/L) | 79.4 ± 41.5 | 97.3 ± 59.4 | 74.1 ± 33.8 | 0.100 |
| Serum white blood cell (/uL) | 6233.3 ± 1788.7 | 6009.1 ± 1204.5 | 6300.0 ± 1937.5 | 0.864 |
| Serum lymphocyte level (/uL) | 1740.6 ± 686.8 | 1631.6 ± 988.6 | 1773.0 ± 582.7 | 0.384 |
| Serum lymphopenia* | 18 (37.5%) | 6 (54.5%) | 12 (32.4%) | 0.288 |
| Serum hemoglobin (g/dL) | 13.7 ± 1.9 | 13.7 ± 1.4 | 13.7 ± 2.0 | 0.749 |
| Serum platelet (x10^3^/uL) | 256.5 ± 81.3 | 263.6 ± 94.9 | 254.4 ± 78.2 | 0.524 |

Data are presented as mean ± standard deviation or frequencies (%). Difference between both groups was analyzed by the chi-square test or Fisher exact test for categorical variables and Mann-Whitney U test for continuous variables. Asterix (*) indicates variables analyzed by Fisher exact test.

Abbreviations: *ACE*, angiotensin-converting enzyme; *LN*, lymph node.
